# Acute Cholecystitis Masking a Gastric Bezoar

**DOI:** 10.7759/cureus.22673

**Published:** 2022-02-28

**Authors:** Manahil Chaudhry, Noreena Iqbal, Ayesha Malik

**Affiliations:** 1 Emergency Medicine, Hameed Latif Hospital, Lahore, PAK; 2 Internal Medicine, Milton Keynes University Trust Hospital, Milton Keynes, GBR; 3 Emergency Medicine, Barking, Havering and Redbridge University Hospitals, London, GBR

**Keywords:** gallbladder disease, acute calculus cholecystitis, delayed gastric emptying, pain abdomen, gastric mass, bezoar

## Abstract

A bezoar is a solid mass of indigestible material that usually forms in the gastrointestinal (GI) tract. Gastric bezoars, located in the stomach, can present variably. They can be asymptomatic or present with GI symptoms like nausea, vomiting, abdominal pain, or more serious complications, such as GI obstruction or perforation. Diagnostic modalities are mainly radiological, and treatment may either be conservative or interventional. Herein, we disclose the case of a 47-year-old female who presented with a two-week history of abdominal pain with an incidental finding of a possible gastric phytobezoar, co-existing with gallbladder disease. Although not previously reported, this study highlights the possibility of an association between gallbladder disease and the formation of a bezoar.

## Introduction

Bezoars are a rare pathological collection of foreign material in the gastrointestinal (GI) tract. The classification of bezoars is based on their prime constituents, namely, phytobezoars which contain non-absorbable food or fibres, lactobezoars which contain mucous or milk products (in infants), trichobezoars which are made of hair, or pharmacobezoars, from medications. Amongst these, phytobezoars are the most commonly occurring [1]. 

Bezoars may be asymptomatic or present with various GI symptoms, for instance, nausea, bloating, and abdominal pain [2]. They may also present with more sinister complications, such as obstruction, bleeding, or perforation [2]. Bezoars primarily form in the stomach and be localised there, or pass into the small bowel. They can even simultaneously exist at multiple sites along the GI tract (GIT) [3]. Treatment may require invasive modalities using endoscopic guidance or dissolution therapy. We report a gastric bezoar diagnosed incidentally in a 47-year-old female patient suffering from acute cholecystitis.

## Case presentation

A 47-year-old female presented to her general practitioner (GP) with abdominal pain for the past two weeks. Her symptoms had worsened in severity over the last one week before presentation. The pain was localised to right upper quadrant with radiation to the right shoulder. There was no history of fever, nausea, vomiting, or altered bowel habits; however, there was generalised itching. Her background history included anxiety and depression for which she was taking selective serotonin re-uptake inhibitors. She admitted to having intentional weight loss of three stones over the past one year, which she managed by keto diet and intermittent fasting.

An outpatient ultrasound (US) abdomen was requested by the GP, which revealed an unexpected finding of "a solid heterogeneous mass of ovoid shape in the epigastric area, measuring 107×58×93 mm. Moderate vascularity was demonstrated during power Doppler study along with an over-distended stomach around the mass". Additionally, "the gall bladder contained multiple calculi casting an acoustic shadow with common bile duct (CBD) of normal shadow and no evidence of calculi". The US abdomen showed no signs of a gastric outlet or intestinal obstruction and there was no mention of any pericholecystic fluid collection. The radiology team flagged the findings and referred the patient to the emergency department from where she was admitted to the acute medical unit (AMU).

On admission, her observations were unremarkable. On inspection, she had generalised scratch marks on her skin but was not jaundiced. Systemic examination revealed a positive Murphy’s sign, while rest of the systemic examination was unremarkable. Routine blood investigations showed alanine aminotransferase 242 IU/L (normal 7-55 IU/L), gamma-glutamyl transpeptidase 650 IU/L (normal 8-60 IU/L), alkaline phosphatase 411 IU/L (normal 30-100 IU/L), total bilirubin of 42 μmol/L (normal 3-17 μmol/L), c-reactive protein 8.1 mg/L (normal <10 mg/L), and serum amylase 101 IU/L (normal 70-300 IU/L). A computed tomography (CT) scan of the abdomen and pelvis was done, which showed a 100×40 mm intraluminal density within the stomach surrounded by fluid and small gas locules in the periphery. These findings were consistent with a gastric bezoar. Furthermore, the CT showed mild thickening of the gall bladder with minimal pericholecystic fluid and a mildly dilated CBD of 8 mm (normal <6-7 mm) (Video 1). 

**Video 1:** A computed tomography (CT) scan of the abdomen and pelvis which shows an intraluminal density (yellow arrow) within the pyloric end of the stomach surrounded by fluid, followed by (selected) successive images.

In light of her diagnosis of acute cholecystitis with a gastric bezoar, input from general surgical and gastroenterology team was sought. During her stay in the AMU, other than symptomatic treatment, she was advised to drink Coca-Cola. While awaiting gastroscopy to further investigate the bezoar, she was given intravenous fluids, intravenous omeprazole, and intravenous antibiotics (amoxicillin + clavulanic acid as per local hospital guidelines). She experienced symptomatic relief over the next few days and on the fifth day of admission a gastroscopy was carried out. The procedure involved scanning till the third part of the duodenum and the study was essentially unremarkable. Despite being seen on two serial imaging modalities, the absence of the bezoar on endoscopy concluded that the gastric bezoar had self-resolved and the constituents likely to be in line with a phytobezoar.

Based on the CT findings of a mildly dilated CBD, she underwent an endoscopic retrograde cholangiopancreatography with a sphincterotomy the same day, which yielded 9-10 small calculi from the CBD. The following day she underwent laparoscopic cholecystectomy, which later identified underlying chronic cholecystitis on histopathology. She was discharged on the ninth day with outpatient general surgery clinic follow-up.

## Discussion

There are several investigation modalities for the diagnosis of bezoar, including abdominal x-ray (AXR), abdominal US, CT, and endoscopy [4]. In a case series by Ripollés et al., AXR was able to identify a bezoar in only about 18% (3/17) of his patients [5]. This could be owing to the variable composition of bezoars which may not always be radiopaque. Hence, US maybe a more sensitive modality to pick up such a finding. On the US, a bezoar appears as a hyperechoic intraluminal mass within the GIT with echogenic components and an intense sonic shadow [6]. In the same case series, Ripollés T. and colleagues found US and CT scan to have a sensitivity of 90% and 100%, respectively [5]. Furthermore, CT images are helpful in recognising complications of bezoars, such as GI obstruction, and can identify if bezoars are present at multiple locations [7]. Finally, endoscopy is the most specific investigation as it involves direct visualisation while offering a therapeutic advantage [4]. In our case, US revealed a solid heterogeneous ovoid shape mass measuring 107×58×93 millimetres with moderate vascularity on the power Doppler, resulting in over-distension of stomach. This was later confirmed to be a gastric bezoar on CT imaging.

The treatment for bezoars depends on the size, location, severity of symptoms, and the associated complications. Smaller bezoars can resolve spontaneously or be dissolved using various medical therapies [8]. One of the commonly used medical agents is carbonated soda. We used Coca-Cola, a popular option in prior literature, and it resulted in resolution of the bezoar. This was evident by a normal endoscopy which took place on day 5. Kramer SJ et al. reported success with dissolution therapy in three patients using Coca-Cola and cellulose [9]. While soda is an effective therapy, its mechanism of action remains disputed. Suggested theories say it could be due to the mucolytic action of the sodium bicarbonate, the bubbling action of carbon dioxide through the bezoar, or the acidity of the cola which has a digestive effect comparable to gastric acid [2]. Other pharmacological treatments include the use of prokinetic agents (e.g. metoclopramide), N-acetylcysteine, or a combination therapy with cellulase, cystine, and metoclopramide [9]. Larger gastric bezoars can be removed effectively by direct endoscopic suction and mechanical fragmentation, or may require surgical exploration [10].

Since Coca-Cola is an effective treatment for phytobezoars [9], which are aggregations of undigested fruit or vegetable fibres, this helps us solve the riddle backwards, suggesting the nature of the bezoar in our case to be a phytobezoar. This bezoar was an incidental finding in our patient who suffered from gallbladder disease. As per our extensive literature reading on bezoars, this is the first case reporting their co-existence, and there might be a likely association. One of the risk factors to bezoar formation is delayed gastric emptying [9]. A few studies in the past have established gallstone disease to be a cause of delayed gastric emptying [11-13]. Ibrarullah et al. hypothesised that this could be due to an effect of hormonal stimuli secreted from inflamed gallbladder wall mucosa that are linked to gastric motility, or due to a pyloroduodenal ileus as a consequence of inflammation in and outside the gallbladder, or even as a result of mechanical obstruction from pericholecystic adhesions secondary to recurrent cholecystitis [11]. On this account, the case adds to the list of rare associated findings with gallbladder disease or vice versa.

## Conclusions

The case references theories from prior literature that propose a link between gallbladder disease and delayed gastric motility. Decreased gastric motility can be a risk factor for the formation of a bezoar. One type is the phytobezoar, composed of indigested intake. While there are endoscopic and surgical options for removing bezoars, this report elaborates an approach to treating gastric bezoars with conservative treatment strategies like dissolution therapy using carbonated drinks such as Coca-Cola. Dissolution therapy can be effective for phytobezoars and should be considered before intervention. 
